# MFNG is an independent prognostic marker for osteosarcoma

**DOI:** 10.1186/s40001-023-01139-x

**Published:** 2023-07-26

**Authors:** Yi Gao, Lili Luo, Yuxing Qu, Qi Zhou

**Affiliations:** Department of Orthopaedics, Changzhou Hospital of Traditional Chinese Medicine Affiliated to Nanjing University of Chinese Medicine, 25 Heping Bei Lu, Tianning District, Changzhou, 213000 Jiangsu China

**Keywords:** *MFNG*, Osteosarcoma, Prognosis, Recurrence, Fringe

## Abstract

**Background:**

Osteosarcoma (OS) has been the most common malignancy of the bone in children and adolescents, and the unsatisfactory prognosis of OS sufferers has long been a hard nut. Here, we delved into the markers with a prognostic value for predicting the prognosis of OS patients.

**Methods:**

The messenger RNA (mRNA) sequencing data and clinical data of OS were retrieved from a Gene Expression Omnibus (GEO) dataset (GSE39058). Next, prognosis-related genes (PRGs) were filtered with the aid of Kaplan–Meier (K-M) curves and Cox regression analysis (CRA). Later, Gene Ontology (GO) biological process analysis was used in verifying the function of different genes. CCK-8 and cell apoptosis assay were performed to evaluate the function of *MFNG* in U2OS cells.

**Results:**

Among the obtained genes, Manic Fringe (*MFNG*) had the closest relevance to prognosis and clinical traits, thus becoming the research object herein. In light of the expression level of *MFNG*, patients fell into high- and low-*MFNG* groups. Patients with elevated *MFNG* expression had a worse prognosis, according to the survival analysis. It was unveiled by the univariate and multivariate analyses that *MFNG* expression was an independent adverse prognostic factor for disease-free survival in OS patients (p = 0.006). Meanwhile, *MFNG* expression was linked to gender and tumor recurrence, and it was higher in patients with OS recurrence. Moreover, overexpression of *MFNG* promoted the cell proliferation and inhibited the cell apoptosis of U2OS cells.

**Conclusions:**

The expression level of *MFNG* negatively correlated with OS progression, and as an independent adverse prognostic factor for disease-free survival in OS patients. Moreover, *MFNG* regulated the cell proliferation and apoptosis of OS cells.

**Supplementary Information:**

The online version contains supplementary material available at 10.1186/s40001-023-01139-x.

## Background

Osteosarcoma (OS), with primitive mesenchymal cells as its origin, is one of the most found primary solid malignancy of the bone, showing an annual prevalence of around 4.8 per million [1–3]. OS, frequently arising in the metaphysis of long bone, features high disability rate. By reason that OS inclines to metastasizing to other sites, especially the lung, OS cases have been plagued by unsatisfactory prognosis [4]. In spite of a 60–70% five-year survival rate of localized OS cases following surgical excision, neoadjuvant chemotherapy and postoperative chemotherapy, the rate was under 30% in OS cases experiencing metastasis [5, 6]. As unveiled by a report, advancement in therapies for OS is limited by the complicated and unstable genome [7], which manifest a necessity for advances in early diagnosis, therapy, and prognosis prediction of OS regarding molecular genetics.

Manic Fringe (*MFNG*) encodes β-1,3-N-acetylglucosaminyltransferase manic fringe that modifies epidermal growth factor repeats in the extracellular domain of Notch [8]. Reports show that *MFNG* functions as an oncogene and exhibits a high expression in claudin-low breast cancer (CLBC) [9]. A lowered *MFNG* level limits neovascularization and the migration of renal carcinoma cells [10]. May wa's team reported that an elevation of *MFNG* was detectable in Ewing's sarcoma cells, and that NIH 3T3 cells with overexpressed *MFNG* display tumorigenicity in mice suffering severe combined immunodeficiency disease [11]. Ewing's sarcoma is one of the most frequently found malignancies of the bone besides OS, usually arise in childhood and adolescence [12, 13]. Nonetheless, the function of *MFNG* in OS prognosis is far from understood.

We found that *MFNG* was an independent prognosis-related gene (PRG) in OS by analyzing the GSE39058. Thus, with the aid of gene expression data in GSE39058, we delved into the interrelation between *MFNG* expression and OS progression, recurrence, and survival. It was unveiled that high *MFNG* expression was indicative of adverse outcomes of OS.

## Methods

### Data acquisition

We retrieved gene expression microarray of OS by simultaneously entering "osteosarcoma" and "survival" in the search box in the gene expression omnibus (GEO, https://www.ncbi.nlm.nih.gov/geo/), and singled out GSE39058 as a training dataset herein. Later, we directly filtered clinical prognostic data from the matrix file of microarray datasets in the GEO database, and the messenger RNA (mRNA) sequencing data were utilized for mRNA profiling.

### Screening of PRGs

Kaplan–Meier (K-M) curves and Cox regression analysis (CRA) were adopted for filtering PRGs by use of "survival" package in R with the thresholds of K-M < 0.01 and p < 0.01 (Additional file 1: Table S1).

### Target gene identification

After screening of PRGs, multivariate CRA was employed to perform independent prognostic analysis (p < 0.01) to obtain genes significantly associated with independent prognosis. Then, we analyzed the correlation between independent PRGs and clinical traits by wilcox.test or kruskal.test. As *MFNG* had the highest correlation with clinical traits, it was selected as the research object.

All OS patients fell into high- (> median, n = 21) and low- *MFNG* groups (≤median, n = 21) *as per* the *MFNG* expression level. Survival analysis of *MFNG* in the prognostic model was implemented with the aid of the K-M curve and log-rank test via the "survival" and "survminer" packages in R.

### Statistical assessment

R-3.5.0 was utilized for assessment of all data. Hypothesis testing in categorical and continuous variables was implemented by the Fisher exact and Wilcoxon rank-sum tests, respectively. The limma package was employed for the assessment of differentially expressed genes (DEGs). Further, the survival was estimated by use of the K-M method and multivariate CRA, and the log-rank test was carried out for intergroup comparisons. Additionally, enriched Gene Ontology (GO) terms were filtered by use of "Clusterprofiler" package.

## Results

### Identification of *MFNG as a target gene*

We got GSE39058 from GEO database (https://www.ncbi.nlm.nih.gov/geo/). *As per* the probe information from Illumina HumanHT-12 WG-DASL V4.0 R2 Expression BeadChip (GPL14951), the annotation of 20,791 genes in 47 OS samples from GSE39058 was annotated. The results of screening of PRGs in OS with K-M curves (KM < 0.01) and CRA (coxPvalue < 0.01) by “survival” R package are shown in Additional file 1: Table S1. After multivariate CRA was adopted for independent prognostic analysis (p < 0.01), we obtained the genes significantly associated with independent prognosis. Next, wilcox.test or kruskal.test (p < 0.05) displayed the correlation between independent PRGs and clinical traits (age,gender,and recurrence) (Table 1). It was uncovered that *MFNG* was associated with two clinical traits (gender and recurrence), so we chose *MFNG* for follow-up study.

**Table 1:** The correlation between independent prognostic-related genes and clinical traits

id	Age	Gender	Recurrence	SigNum
MFNG	0.588539	0.030243	0.024712	2
C11orf82	0.018869	0.93052	0.122259	1
GNA15	0.791803	0.089273	0.044442	1
IL4R	0.005387	0.348698	0.276478	1
C2CD3	0.294969	0.574711	0.141906	0
INO80E	0.125041	0.122259	0.417065	0
LGR4	0.504687	0.81289	0.056665	0
MIR1255B1	0.285712	0.37126	0.08448	0
ROBLD3	0.184119	0.402807	0.13511	0
SHANK3	0.101548	0.736545	0.084531	0
WDR1	0.13824	0.388839	0.402807	0

### Survival outcomes in high-*MFNG* group and low-*MFNG* group

**Fig. 1:**
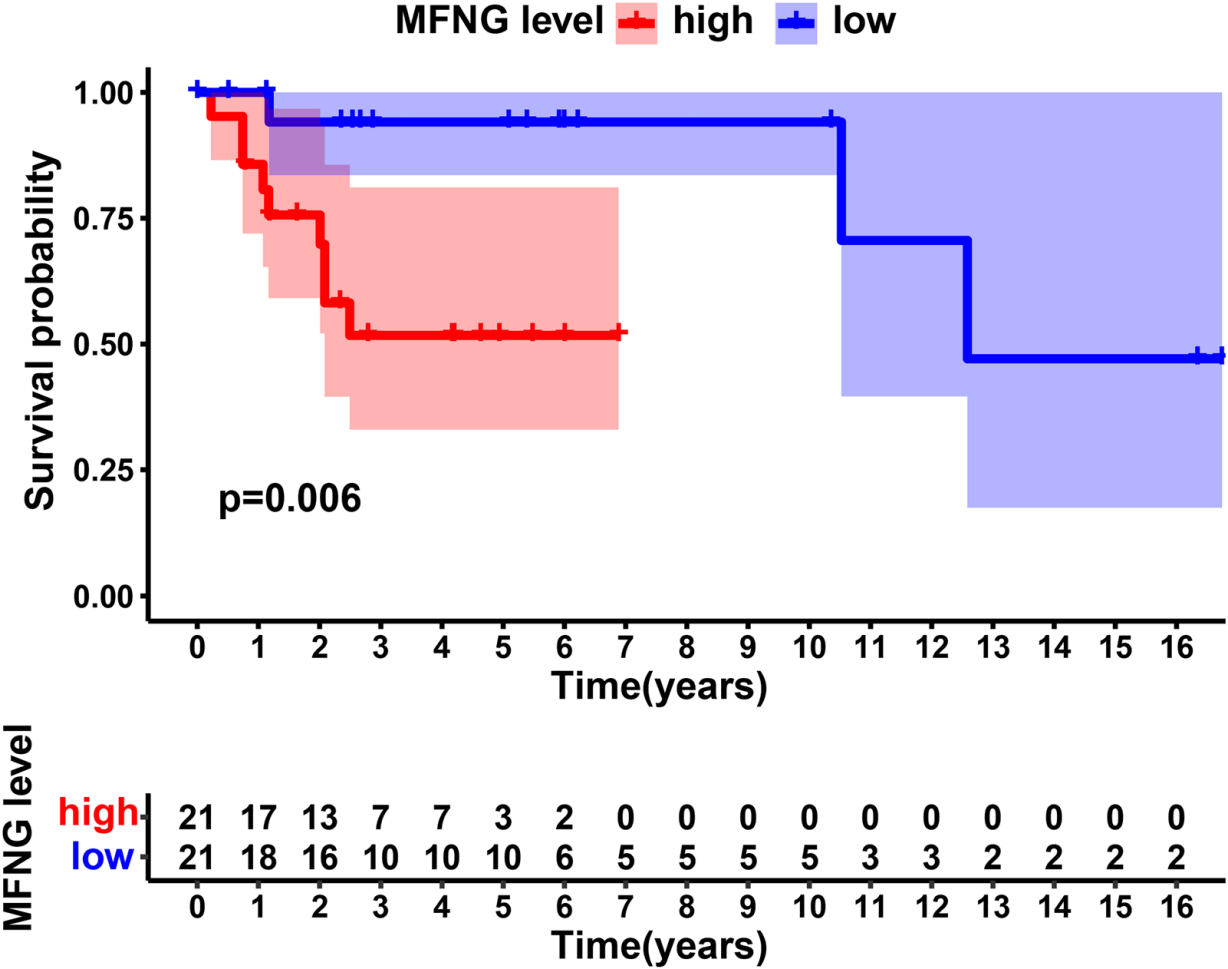
Survival outcomes in high-*MFNG* group and low-*MFNG* group. Osteosarcoma patients in *MFNG* high group had worse outcome than that in *MFNG* low group

All OS patients fell into high- (> median, n = 21) and low- *MFNG* groups (≤median, n = 21) *as per* the *MFNG* expression level. Survival analysis of *MFNG* in the prognostic model was implemented with the aid of the K-M curve and log-rank test via the "survival" and "survminer" packages in R. As shown in Fig. 1, OS patients in high-*MFNG* group had worse outcome than those in low-*MFNG* group.

**Fig. 2:**
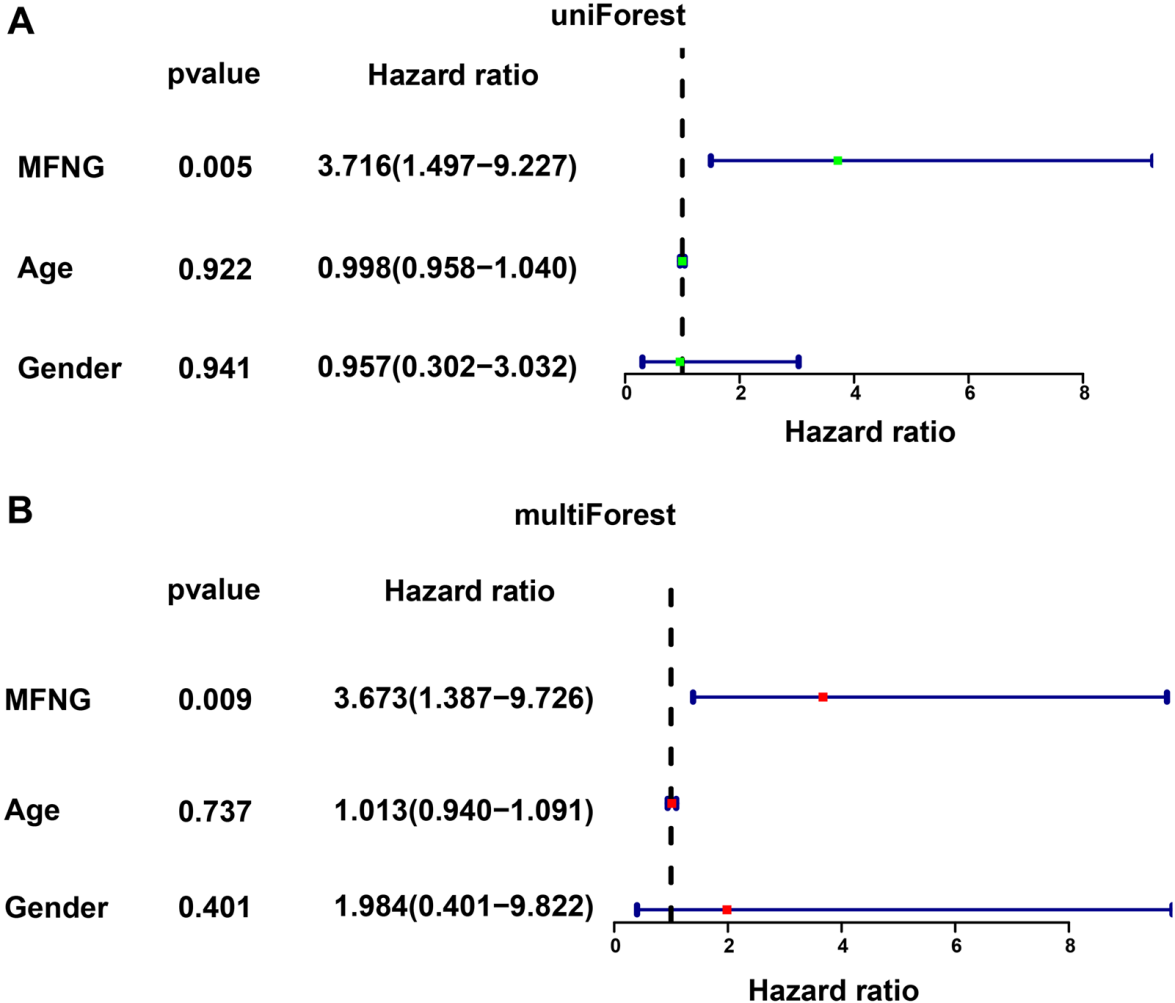
Cox regression analysis in high-*MFNG* group and low-*MFNG* group. **A** Univariate Cox regression analysis and **B** multivariate Cox regression analysis screened out the independent prognostic-related factor

In univariate and multivariate CRA, *MFNG* exhibited notable relevance to the prognosis of OS and was proven to be an independent prognostic factor (PF) (Fig. 2A, B; Additional file 2: Table S2).

### Differences in clinical data of OS cases between high-*MFNG* group and low-*MFNG* group

**Fig. 3:**
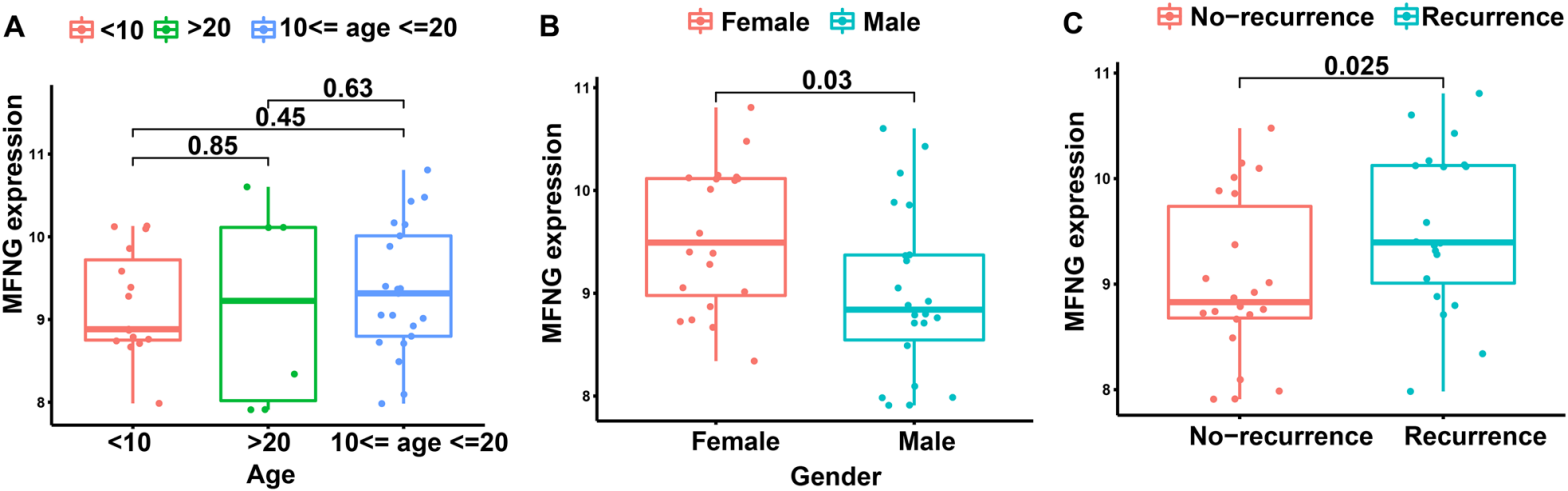
Differences in clinical data of OS cases between high-*MFNG* group and low-*MFNG* group. Subgroups analysis of *MFNG* in accordance with different **A** age, **B** sex, and **C** recurrence

A report shows that worldwide, 15–19-year-old boys and 10–14-year-old girls displayed the highest OS prevalence, in line with age of puberty [14]. Age groups include < 10-year-old group, 10–20-year-old group, and > 20-year-old group. In the current study, the differences in clinical data of OS cases between high-*MFNG* group and low-*MFNG* group were analyzed through "ggpubr" package. There was no significant difference in the age between high-*MFNG* group and low-*MFNG* group (Fig. 3A), while there were significant differences in the gender and recurrence (Fig. 3B, C). It can be concluded that male OS patients have a higher tendency of low *MFNG* expression, and OS patients with no recurrence have a higher tendency of low *MFNG* expression.

### Analysis of DEGs and pathways in high-*MFNG *group versus low-*MFNG* group

**Fig. 4:**
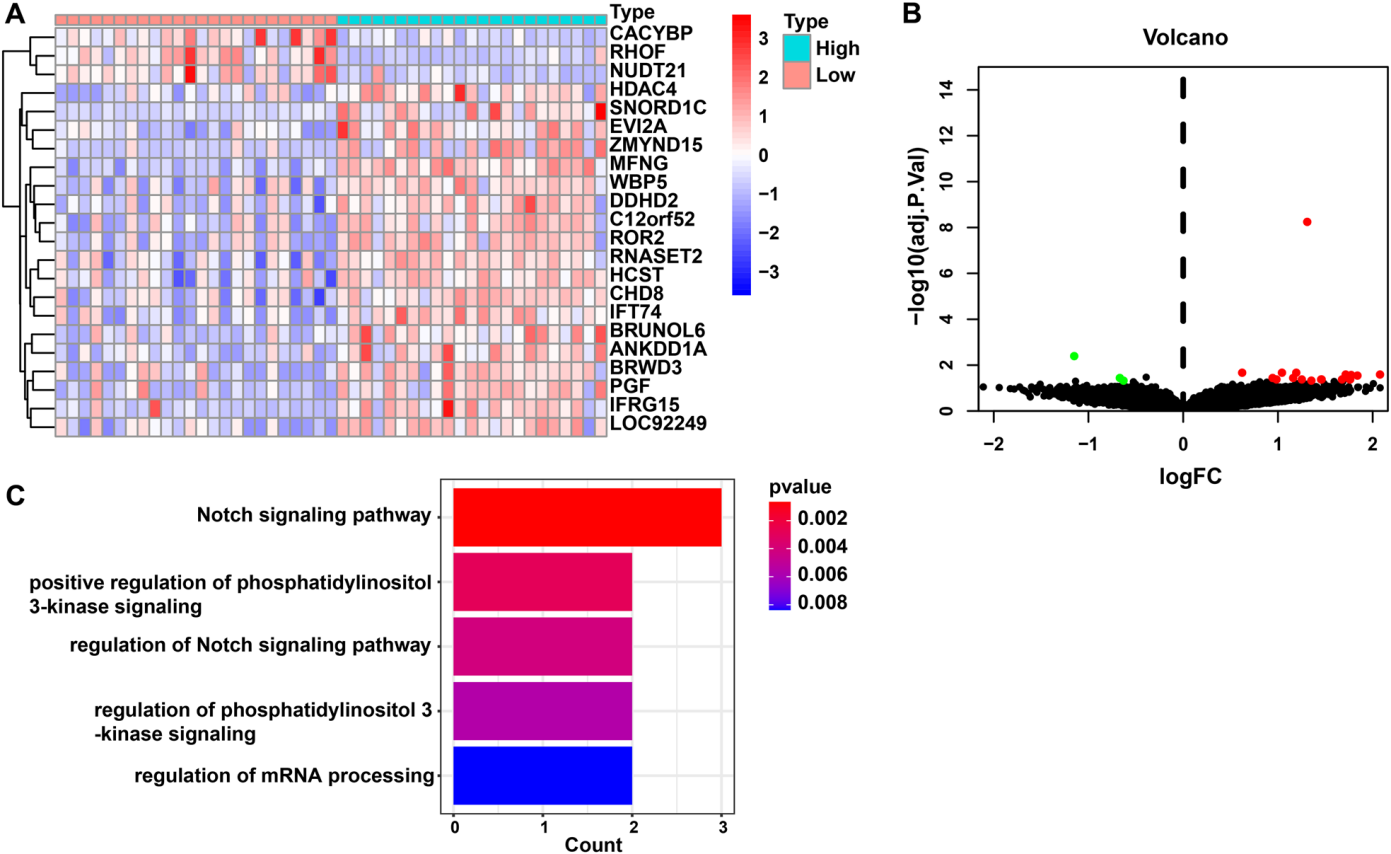
Analysis of DEGs and pathways in high-*MFNG* group versus low-*MFNG* group. **A** Heatmap and **B** volcano plot show DEGs between high-*MFNG* group versus low-*MFNG* groups. Cut-off criteria for DEGs significance was adj.P < 0.05 and the absolute value of the log2 fold change > 0.5. **C** GO result for differential expression genes

With the aim for determining *MFNG*-associated genes, the expression of DEGs was compared between high-*MFNG* group and low-*MFNG* group. The results showed 19 up-regulated genes and 3 down-regulated genes (adj.P < 0.05, (FC, log2) > 0.5 or < -0.5, Fig. 4A, B, Additional file 3: Table S3). Later, the enriched GO terms were assessed by use of DEGs. It was uncovered that a majority of DEGs were enriched in GO terms involving the biological process, including Notch signaling pathway (GO:0007219), positive regulation of phosphatidylinositol 3-kinase signaling pathway (GO:0008593), regulation of Notch signaling pathway (GO:0008593), regulation of phosphatidylinositol 3-kinase (PI3K) signaling pathway (GO:0014066) and regulation of mRNA processing (GO:0050684) (Fig. 4C, Additional file 4: Table S4).

### Module screening from the protein–protein interaction (PPI) network

**Fig. 5:**
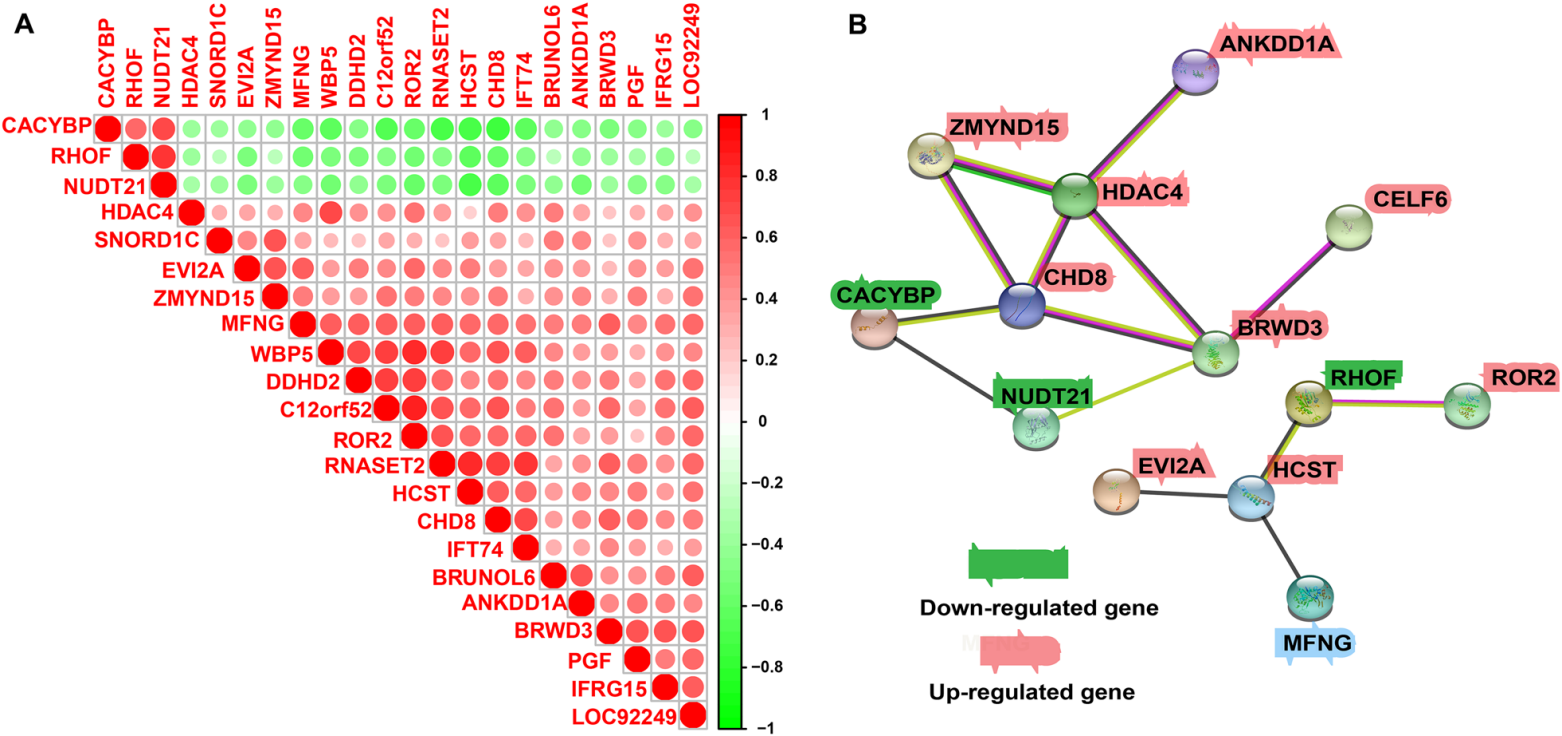
Module screening from the PPI network. **A** The correlation analysis of DEGs with the Pearson correlation coefficient. **B** PPI network of top DEGs

We measured the correlation among the top 22 DEGs in high-*MFNG* group and low-*MFNG* group (Fig. 5A). Additionally, the PPI network of 22 DEGs was built with the use of the String database, which unveiled interactions among most of the up-regulated genes and all down-regulated genes (Fig. 5B).

### *MFNG* promotes the cell proliferation and inhibits the cell apoptosis of U2OS cells

**Fig. 6:**
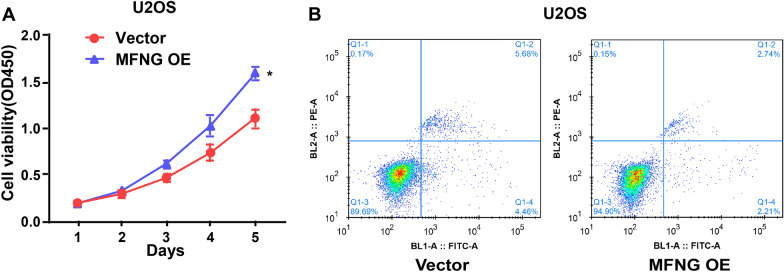
*MFNG* promotes the cell proliferation and inhibits the cell apoptosis of U2OS cells. **A** CCK-8 detected the proliferation of U2OS cell after transfection. **B** Cell apoptosis of U2OS cell after transfection was detected. *P < 0.05

In Fig. 5B, we can see that hematopoietic cell signal transducer (HCST) is co-expression with *MFNG.* Reports showed that HCST affects cell proliferation and survival by participating in the natural killer and T cell responses and in the activation of PI3K-dependent signaling pathway [15]. Thus, we want to explore the role of *MFNG* in OS cells. We overexpressed *MFNG* in OS cell line U2OS and found that *MFNG* overexpression (*MFNG* OE) promoted the cell proliferation and inhibited the cell apoptosis of U2OS cells (Fig. 6A, B).

## Discussion

OS is the most found malignancy of the bone arising in childhood and adulthood, showing a high tendency of invasion and metastasis [1, 16, 17]. Since 2000, threefold elevation of the OS incidence rate has been observed in 0–24-year-old cases [2]. Up-to-now therapies for OS patients are neoadjuvant chemotherapy, surgery, radiotherapy and chemotherapy [18, 19]. Nonetheless, the high malignancy of OS results in poor overall survival of OS patients, especially those in the advanced stage [2, 20, 21]. Hence, in-depth deciphering of the molecular pathomechanism by which OS occurs and progresses appears to be a necessity, which benefits filtering of pivotal molecules or biomarkers for early diagnosis, targeted therapy, and prognosis assessment of OS.

Our research demonstrated that lower *MFNG* expression in OS cases showed relevance to satisfactory prognosis and low recurrence. *MFNG* has also been reported to be associated with the recurrence of ovarian carcinoma [22]. As unveiled by a study of Zhang et al*.*, a high *MFNG* expression was detectable in CLBC and served as an oncogene by activating Notch signaling, thus hastening tumor cell migration, tumorsphere formation, and epithelial-to-mesenchymal transition (EMT) [9]. Beyond that, *MFNG* turns out to be a determinant player in optimal B and T cell development. In detail, it leverages a facilitating role in Th1 cell development and a suppressing role in Th2 cell development [23, 24].

Likewise, enrichment of the GO pathways is mainly detectable in the Notch signaling pathway and regulation of PI3K signaling pathway. Notch signaling pathways are a class of highly conserved signaling pathways in multicellular organisms that mediate the influence and regulation of the external environment on cells by participating in intercellular interactions, and programmatically manipulate cell fate and tissue differentiation in the early development of organisms [25]. Notch signaling pathways modulate cell invasion, adhesion, proliferation, apoptosis, and differentiation by virtue of cell–cell interactions [26, 27]. Beyond that, the growth and progression of human malignant tumors including OS appear to be subjected to influences by the Notch signaling pathway [28, 29]. For example, the Notch signaling pathway probably drives the differentiation of bone marrow mesenchymal stem cells into osteoblasts, thus facilitating ectopic bone formation [30]. *DLX5* activates the Notch signaling pathway to benefit OS progression [31]. Suppressing the Notch signaling pathway put a brake on the progression of cell motility, metastasis, and EMT-like phenomena resulting from low-level cisplatin in OS [32]. Overexpression of lncRNA *CEBPA-AS1* keeps a rein on OS cell proliferation and migration and elicits their apoptosis via the Notch signaling pathway [33]. On the other hand, activating the Notch signaling pathway can control the differentiation of macrophages into M1 and benefits inflammation and antitumor activity, but impeding the Notch signaling pathway polarizes macrophages to M2, so as to hinder inflammation and drive tumor growth [34].

PI3K, a family of lipid kinases, modulates a cascade of physiological cell processes such as metabolism, motility, exocytosis differentiation, proliferation, apoptosis, exocytosis and autophagy [35]. Subsequent to activation at the cell surface, the PI3K signaling pathway integrates signals from cytokines, growth factors and environmental signals and delivers them to effector molecules controlling protein synthesis, growth, survival and proliferation via protein kinase B (AKT) and mammalian target of rapamycin (mTOR) [36, 37]. AKT is the vital mediator of the PI3K signaling pathway, and its abnormal activation is interrelated to a multifold of malignant tumors [38, 39], including OS. For example, *LINC00968* activates the PI3K/AKT/mTOR signaling pathway to exert an oncogene action [40]. PI3K inhibitors impair tumor progression and enhance sensitivity to anlotinib in anlotinib-resistant OS [41]. *MDA19* inhibits OS via suppressing the PI3K/Akt/mTOR signaling pathway [42].

The PPI network also revealed that hematopoietic cell signal transducer (HCST) was co-expressed with *MFNG*. Reports show that HCST participates in the activation of PI3K-dependent signaling pathway and in the natural killer and T cell responses, which affect cell survival and proliferation [15]. Besides, HCST is interrelated to the poor prognosis of clear cell renal cell carcinoma [43]. We also performed CCK-8 and cell apoptosis assay in U2OS cells which were transfected with vector or *MFNG* OE. We found that *MFNG* OE promoted the cell proliferation and inhibited the cell apoptosis of U2OS cells.

However, there still some limitations in our study. Firstly, the current research is only based on public databases, we will further collect clinical specimens to verify our conclusion. Secondly, the specific physiopathologic mechanism developed by *MFNG* to function in OS cells has not yet been fully understood. In the future, we will conduct more detailed molecular mechanism research and gain a deeper understanding of *MFNG* at the OS genome level.

## Conclusions

In closing, the low *MFNG* expression is a good PF in OS cases, while the high *MFNG* expression is associated with recurrence of OS. Additionally, enriched GO terms and PPI networks pertinent to OS shed light on the delving into the pathogenesis of high and low *MFNG* expressions.

## Supplementary Information

**Additional file 1. Table S1.** The prognosis-related gene in OS.

**Additional file 2. Table S2.** The univariate and multivariate Cox regression analysis.

**Additional file 3. Table S3.** The differentially expressed genes in high-*MFNG* group versus low-*MFNG* group.

**Additional file 4. Table S4.** GO result for differential expression genes.

## Data Availability

Not applicable.

## Abbreviations

OS: Osteosarcoma
GEO: Gene expression omnibus
K-M: Kaplan–Meier curve
PRG: Prognosis-related gene
CRA: Cox regression analysis
GO: Gene ontology
MFNG: Manic fringe
